# Rationale and Means to Target Pro-Inflammatory Interleukin-8 (CXCL8) Signaling in Cancer

**DOI:** 10.3390/ph6080929

**Published:** 2013-08-06

**Authors:** Laura M. Campbell, Pamela J. Maxwell, David J.J. Waugh

**Affiliations:** Centre for Cancer Research and Cell Biology, Queen’s University Belfast, 97 Lisburn Road, Belfast, Northern Ireland, BT9 7BL, UK; E-Mails: lcampbell43@qub.ac.uk (L.M.C.); pamela.maxwell@qub.ac.uk (P.J.M.)

**Keywords:** cancer, inflammation, cxcl8, cxcr1, cxcr2, chemokine-targeted therapy

## Abstract

It is well established that chronic inflammation underpins the development of a number of human cancers, with pro-inflammatory signaling within the tumor microenvironment contributing to tumor progression and metastasis. CXCL8 is an ELR^+^ pro-inflammatory CXC-chemokine which mediates its effects via signaling through two G protein-coupled receptors, CXCR1 and CXCR2. Elevated CXCL8-CXCR1/2 signaling within the tumor microenvironment of numerous cancers is known to enhance tumor progression via activation of signaling pathways promoting proliferation, angiogenesis, migration, invasion and cell survival. This review provides an overview of established roles of CXCL8-CXCR1/2 signaling in cancer and subsequently, discusses the possible strategies of targeting CXCL8-CXCR1/2 signaling in cancer, covering indirect strategies (e.g., anti-inflammatories, NFκB inhibitors) and direct CXCL8 or CXCR1/2 inhibition (e.g., neutralizing antibodies, small molecule receptor antagonists, pepducin inhibitors and siRNA strategies). Reports of pre-clinical cancer studies and clinical trials using CXCL8-CXCR1/2-targeting strategies for the treatment of inflammatory diseases will be discussed. The future translational opportunities for use of such agents in oncology will be discussed, with emphasis on exploitation in stratified populations.

## 1. Introduction

It is now widely accepted that inflammation is a major contributor to the development and progression of many human cancers, with inflammation now identified as the seventh hallmark of cancer [1]. A number of chronic inflammatory conditions are known to significantly increase the risk of developing particular cancers, with approximately 20% of all cancers believed to result from chronic inflammation or inflammatory states [2]. For instance, inflammatory bowel disease is associated with an increased risk of colon cancer development [3,4] and chronic prostatitis has been linked to the development of prostate cancer [5,6]. Furthermore, regular long-term use of anti-inflammatory drugs (e.g., aspirin) is known to decrease the risk of several cancer types [7,8]. A possible mechanism underlying this extrinsic cancer-related inflammation may involve the induction of DNA damage by reactive oxygen and nitrogen species produced by infiltrating inflammatory cells during chronic inflammation [9]. Alternatively, intrinsic cancer-related inflammation may be induced as a result of the genetic or epigenetic changes responsible for cellular transformation [10]. This is due to the activation of downstream signaling pathways capable of mediating inflammatory responses. For example, oncogenic activation of Ras results in the up-regulation of key pro-inflammatory chemokines and cytokines due to activation of MAPK and PI3K signaling pathways [11,12,13]. Likewise, loss of the PTEN tumor suppressor gene has recently been shown to potentiate CXC-chemokine signaling in prostate cancer [14]. Such pro-inflammatory signaling is not restricted to the tumor cells themselves, but is reinforced by paracrine signaling to and from infiltrating inflammatory cells, stromal fibroblasts and endothelial cells of the tumor microenvironment [15]. Inflammation in the tumor microenvironment promotes proliferation and survival of tumor cells, angiogenesis and metastasis [16]. Furthermore, the presence of inflammation also promotes resistance to hormonal therapies and chemotherapeutic agents [17,18]. Cancer-related inflammation may therefore be a valuable target for therapeutic intervention.

Interleukin-8 (IL-8) or CXCL8 is a pro-inflammatory ELR^+^ CXC-chemokine, originally identified as a neutrophil chemoattractant [19]. CXCL8 makes an important contribution to the induction of innate immunity, through its effects on neutrophil chemotaxis and activation [20]. Accordingly, CXCL8 has been implicated in a number of inflammatory diseases, including inflammatory bowel disease [21,22] and rheumatoid arthritis [23]. CXCL8 is secreted from a range of cell types including leukocytes, fibroblasts, endothelial cells and malignant cancer cells. It is synthesized as an inactive 99 amino acid precursor protein, with N-terminal cleavage producing a 77 (fibroblasts and endothelial cells) or 72 (leukocytes) amino acid protein. The 77 amino acid isoform secreted from non-immune cells is further cleaved following secretion to produce the active 72 amino acid CXCL8 protein, with a molecular weight of approximately 8kDa [24,25]. Furthermore, CXCL8 can exist in monomer or dimer forms, which are capable of differentially activating and regulating its two cell surface receptors [26].

CXCL8 mediates its effects via binding to two heterotrimeric G protein-coupled receptors, CXCR1 and CXCR2. These receptors are normally found on the surface of leukocytes (neutrophils, monocytes, macrophages, basophils, T-lymphocytes) and endothelial cells. However, they are also known to be expressed on the tumor cells and tumor-associated stromal cells of various cancers. The two receptors share 78% homology. However, differences in their N-terminal domains result in different binding specificities [27]. CXCR1 binds CXCL6 and 8, while the “promiscuous” CXCR2 receptor has high binding affinity for CXCL1, 2, 3, 5, 6, 7 and 8 [28], as well as macrophage inhibitory factor (MIF), a novel ligand for CXCR2 [29]. Ligand binding is proposed to occur at the juxta-membrane region of the receptor, with involvement of residues resident within the extracellular loops with further contributions from hydrophobic core of the receptor, which is formed by the seven transmembrane α-helices [30]. Binding of ligand to the receptor induces conformational changes in the intracellular loops and C-terminal domain, exposing epitopes required for heterotrimeric G protein coupling. The classic chemotactic CXCL8 response involves activation of pertussis toxin-sensitive Gαi-proteins [31,32]. However, other non-classical CXCL8 responses are thought to involve activation of pertussis-insensitive Gα-proteins [33]. G protein activation occurs following exchange of GDP to GTP, leading to dissociation of the α and βγ subunits of the G protein, which can subsequently modulate downstream effectors, initiating signaling cascades [30].

Following ligand-induced activation, CXCR1/2 becomes phosphorylated, desensitised and internalised. Studies have shown that CXCR2 is internalised more rapidly than CXCR1, and its re-expression on the cell surface occurs more slowly [34]. This internalisation and recycling of the receptors plays a major role in the regulation of CXCR1/2 signaling. A range of signaling pathways may be activated downstream of CXCR1/2, including MAPK, PI3K, PKC, FAK and Src. Reports from various groups have demonstrated that CXCL8 signaling activates multiple transcription factors such as NFκB, AP-1, HIF-1 and STAT3, and in prostate cancer cells, this signaling has been shown to induce activation of the androgen receptor (AR) [35].

**Figure 1:**
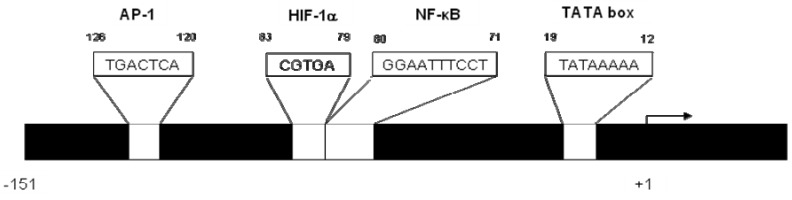
Schematic diagram illustrating the potential mechanisms that can underpin transcription of the IL-8 gene.

CXCL8 signaling is normally under tight regulation, with minimal CXCL8 and CXCR1/2 expression in normal tissue [36]. CXCL8 signaling is known to be induced by inflammatory signals (e.g., TNF-α and IL-1), reactive oxygen species, death receptors and steroid hormones (e.g., androgens) [35,37,38]. CXCL8 signaling may also be induced as a result of numerous environmental stresses within the tumor microenvironment including hypoxia, acidosis, hyperglycemia, cytotoxic chemotherapies and radiation [37]. Additionally, tumor cells harboring oncogenic mutations or loss of tumor suppressor genes that lead to the activation of specific signaling pathways have been shown to regulate CXCL8 or CXCR1/2 expression. For example, activating mutations in Ras-GTPase or loss of functional PTEN, can result in constitutive CXCL8 signaling [11,12,14]. The effects of oncogenes and tumor suppressor genes may be much broader since the CXCL8 promoter contains consensus binding sites for NFκB, AP-1, C-EBP, β-Catenin/Tcf and HIF-1 transcription factors (Figure 1) [36,39,40]. Understanding the correlation between oncogenes, tumor suppressors and the regulation of CXCL8 (or CXCL1, CXCL5 or CXCL6) expression will have important consequences for developing stratified or personalized therapeutic strategies wherein the genetic background of tumors can be used to select appropriate patients in which the exploitation of CXCL8 signaling-inhibitors is most likely to show clinical benefit.

The regulation of CXCL8 expression mainly occurs at the transcriptional level, however, post-transcriptional regulation can occur via stabilization of mRNA transcripts (involving the p38 MAPK pathway), with steady-state mRNA levels being proportional to CXCL8 secretion [36,37,41,42]. There is also a small body of evidence supporting translational regulation of CXCL8 [43,44], with CXCL8 synthesis in Ca^2+^ ionophore-treated neutrophils partially suppressed by cycloheximide, but not actinomycin D [45].

## 2. CXCL8 Signaling in Cancer

In addition to its physiological roles in neutrophil chemotaxis, CXCL8 has been implicated in many of the pathological processes involved in cancer progression. Elevated expression of CXCL8 and its receptors has been detected in many types of cancer including prostate [46], colorectal [47], and non-small cell lung cancer [48]. Focusing on prostate cancer, the major interest of our laboratory, Veltri and colleagues initially confirmed that serum CXCL8 levels correlated with prostate cancer stage, with the greatest levels detected in patients with metastatic prostate cancer [49]. Moreover, we have demonstrated an increase in the expression levels and an altered distribution of CXCL8 and its two receptors, CXCR1 and CXCR2, in prostate biopsy tissue; the altered distribution was consistent with prostate cancer cells being subject to an increased autocrine stimulus, and the intensity of expression was observed to increase from high grade prostatic intra-epithelial neoplasia (PIN) through to castrate-resistant prostate cancer [46].

The biochemical relationship of CXCL8 signaling and prostate cancer progression has been studied extensively in *in vitro* and *in vivo* models. The dependence on androgen signaling is a major drive for prostate cancer progression. Using *in vitro* models, we characterized the role of CXCL8 signaling in driving the transition to an androgen-independent, more appropriately known as castrate-resistant state. CXCL8 was observed to induce AR expression and activity, in an androgen-independent manner and promote the proliferation of androgen-dependent LNCaP and 22Rv1 cell lines under androgen-depleted conditions [50]. The ability of CXCL8 to promote progression to this castrate-resistant state has been verified by several additional groups [51,52]. Moreover, we have shown that CXCL8 signaling can regulate the proliferation of castrate-resistant cells by alternative mechanisms, including the capacity to regulate the translation and expression of oncogenes. Studies in two androgen-independent models, PC3 and DU145 cells, confirmed that CXCL8 signaling can up-regulate cyclin D1 expression promoting tumor cell proliferation [53]. This rapid induction of cyclin D1 expression was mediated by the combined activities of CXCL8-promoted Akt/mTOR and MAPK signaling resulting in the activation of the translational machinery. CXCL8 is not only known to promote the proliferation of prostate cancer cells; studies from other laboratories have demonstrated CXCL8-induced proliferation in colon [54], non-small cell lung cancer [55] and melanoma cell lines [56].

The growth and metastasis of prostate cancer is also highly dependent on angiogenesis. The ability of CXCL8 to mediate angiogenesis in many cancer types is well established [57]. An *in vivo* study by Kim *et al.* eloquently demonstrated the major roles played by CXCL8 in promoting the angiogenesis and metastasis of human prostate cancer cells implanted orthotopically in nude mice [58]. High CXCL8 secreting PC3 clones were shown to produce highly vascularized prostate tumors, with a significantly higher rate of lymph node metastases than that of PC3 clones secreting low levels of CXCL8. This study also showed elevated levels of numerous genes involved in angiogenesis and metastasis, including VEGF, MMP-2 and MMP-9 in the high CXCL8 clones. Moreover, a study by Moore *et al*. showed that neutralizing CXCL8 antibodies are capable of inhibiting tumor growth and reducing angiogenic activity of PC3 xenograft tumors in SCID mice [59]. CXCL8 or CXCR1/2-promoted angiogenesis has also been observed in numerous other cancer types, including melanoma [60], pancreatic [61], colon [54], and non-small cell lung cancer [62]. Furthermore, there is evidence to support a role of CXCL8 signaling in promoting the migration and invasion of cancer cells. CXCL8 has been shown to induce the migration of PC3 cells on laminin, and drive invasion through a reconstituted basement membrane [63]. Additionally, CXCL8 has been shown to induce the migration of androgen-dependent LNCaP cells, via activation of Src and FAK [52]. Unpublished observations from our group also support the biochemical association of CXCL8 with Src and FAK-induced chemotaxis and adhesion to bone marrow endothelial cells (a common clinical site of extravasation), while studies on prostate cancer biopsy tissue demonstrate that expression of CXCL8 correlates with increased FAK and Src phosphorylation [64]. CXCL8 signaling has also been shown to promote migration and invasion in melanoma [65], colon [66] and gastric cancer [67].

Neutrophil chemotaxis and activation are major physiological roles of CXCL8 [20]. It is now widely accepted that neutrophil recruitment to the tumor site can facilitate tumor progression [68], with particularly well established roles in tumor angiogenesis [69]. A range of clinical studies have correlated intratumoral neutrophils with poor prognosis in multiple cancer types, including melanoma [70], colorectal [71] and renal cell carcinoma [72]. Additionally, numerous *in vitro* and *in vivo* studies have elucidated the role of neutrophils in the progression of multiple cancer types. For instance, breast cancer cells have been shown to stimulate oncostatin M release from neutrophils, which in turn increased invasive potential of the breast cancer cells [73]. Additionally, tumor-associated neutrophils have been shown to be crucial for colitis-associated carcinogenesis in mice, thought to involve neutrophil expression of MMP-9 and neutrophil elastase [74]. Moreover, it has been shown that impeding neutrophil recruitment to the tumor site via CXCL8 or CXCR1/2 inhibition can reduce tumor growth *in vivo*. Tazzyman *et al*. demonstrated that the use of a CXCR2 antagonist (AZ10397767) was capable of impeding neutrophil infiltration both *in vivo* and *in vitro*, which was associated with reduced tumor growth [75]. Additionally, a study by Farooq *et al.* showed that CXCR2^−/−^ or anti-CXCR2 antiserum-treated mice had lower symptom scores for DSS-induced colitis, with significantly lower polymorphonuclear neutrophil (PMN) infiltration [76]. Similarly, Jamieson *et al.* showed that pepducin-mediated CXCR2 inhibition reduced spontaneous benign tumor formation in APC^Min/+^ mice, with a concurrent reduction in myeloperoxidase (MPO)^+^ cells [77]. CXCR1/2-targeted therapies may therefore reduce intratumoral neutrophils, thereby impeding tumor progression facilitated by neutrophil infiltration.

**Figure 2:**
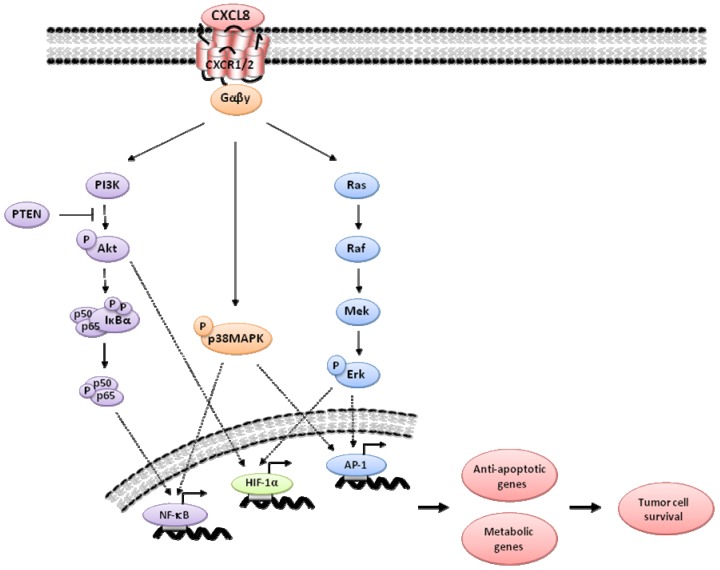
Schematic diagram illustrating the principal signaling pathways activated by IL-8 and their relevance to cell survival. IL-8 induced promotion of NF-κB, HIF-1 or AP-1 transcription factors can increase expression of genes associated with the regulation of apoptosis or cellular metabolism, permitting survival and adaptation in stressful environments.

CXCL8 signaling has also been shown to have an emerging importance in promoting cell survival, by driving anti-apoptotic gene expression (Figure 2). This is especially evident in the context of environmental or treatment-induced stresses. Although other groups had previously characterized that hypoxia induces CXCL8 expression, we showed that hypoxia also induced CXCR1 and CXCR2 expression via HIF-1 and NFkB activation, resulting in an increased CXCL8-signaling stimulus in hypoxic cells. Interestingly, we showed that this stress-induced CXCL8 signaling underpinned the intrinsic resistance of hypoxic cells to the DNA damage chemotherapy agent, etoposide [78]. Subsequently, our group demonstrated that autocrine CXCL8 signaling confers resistance to the DNA-damaging agent oxaliplatin, the death receptor agonist TRAIL and anti-metabolites in prostate cancer cells [79,80,81]. In each case, administration of the anti-cancer agent was shown to induce CXCL8 expression and secretion, as well as expression of the CXCR1 and CXCR2 receptors. CXCL8-mediated chemoresistance to oxaliplatin was shown to be driven by induction of NFkB-transcription, resulting in the up-regulation of multiple anti-apoptotic genes, including Bcl-2 and survivin. Co-administration of a CXCR2 antagonist (AZ10397767) with oxaliplatin resulted in attenuation of NFkB activation, down-regulation of anti-apoptotic gene expression and the potentiation of oxaliplatin cytotoxicity in these cells [79]. In a further study, resistance to TRAIL was shown to result from a CXCL8-mediated transcriptional up-regulation of the endogenous caspase-8 inhibitor, c-FLIP [80]. Both c-FLIP isoforms, c-FLIP_L_ and c-FLIP_S_, were up-regulated by CXCL8 in prostate cancer cells. Inhibition of CXCL8 signaling or down-regulation of c-FLIP sensitized prostate cancer cells to tumor necrosis factor-related apoptosis-inducing ligand (TRAIL)- and chemotherapy (oxaliplatin)-induced apoptosis in both androgen-dependent (LNCaP) and androgen-independent (PC3) cell lines. We have also demonstrated the ability of the CXCR2 antagonist, AZ10397767, to sensitize PC3 cells to the anti-metabolite agents 5-fluorouracil and Tomudex^®^ (ralitrexed) [81], however, of major clinical relevance, we have shown that the inhibition of CXCR2 signaling potentiates the activity of the AR antagonist bicalutamide [50]. The role of CXCL8-CXCR1/2 signaling in chemoresistance has also been well characterized in colorectal cancer, where CXCL8 has been shown to reduce sensitivity to oxaliplatin *in vitro* [54]. Furthermore, a CXCR2 antagonist (SCH527123) has been shown to sensitize cells to oxaliplatin *in vivo* [82]. CXCL8 signaling has also been associated with an increased resistance to cisplatin and paclitaxel in ovarian cancer [83]. This body of research supports the principal use of CXCR1/2-targeted therapeutics as chemo-modulators, acting to down-regulate stress-induced survival pathways in malignant cells with the effect of potentiating the cytotoxicity of conventional and non-conventional chemotherapeutic agents.

There is an extensive body of evidence to support the role of CXCL8-CXCR1/2 signaling in promoting tumor progression. Targeting this signaling network may therefore impede tumor progression by retarding cell proliferation, preventing angiogenesis, down-regulating migration and invasion, and abrogating key mechanisms of cell survival and chemoresistance. The remainder of this review will focus on elucidating some of the principal strategies available for targeting CXCL8-CXCR1/2 signaling in cancer, both indirectly with the use of anti-inflammatories, and directly, using CXCL8- or CXCR1/2-targeted inhibitors. Data obtained from both pre-clinical cancer studies, and clinical trial data acquired from other inflammatory diseases will be presented, followed by the discussion of a number of key translational issues pertinent to use of such targeting strategies in malignant disease, including the relevance of patient stratification and the impact of any potential toxicity/side effects.

## 3. Indirect Targeting

CXCL8 signaling may be targeted not only through the direct regulation of CXCL8/CXCR1/CXCR2 expression and signaling, but also indirectly, through the regulation of key signaling pathways and transcription factors responsible for the regulation of CXCL8 and CXCL8 receptor expression. CXCL8 production is regulated by a combination of three mechanisms: derepression of the gene promoter; transcriptional activation of the gene through activation of the transcription factors HIF-1, AP-1 and NFκB [40,84,85]; and stabilization of the mRNA by the p38 MAPK pathway [86]. Strategies to target these transcription factors and signaling pathways may therefore attenuate CXCL8 signaling in cancer cells indirectly, thereby sensitizing cancer cells to conventional therapeutic interventions.

### 3.1. Signal Transduction Pathway Inhibitors

Virtually all stimuli known to induce CXCL8 production activate a number of protein kinases, which in principal have the ability to modulate NF-κB or AP-1 activity. Activation of these transcription factors will, in turn, promote the transcription of the CXCL8 gene. Therefore, agents that target these transcription factors may, indirectly, also target tumor-derived CXCL8.

#### 3.1.1. MAPK Inhibitors

The transcription factor AP-1 plays a major role in the regulation of CXCL8 transcription and is activated by MAPK signaling. Three MAPK pathways contribute to CXCL8 gene expression; the ERK, JNK, and p38 MAPK cascades. While the ERK and JNK pathways contribute to CXCL8 regulation through transcriptional mechanisms, the p38 MAPK pathway contributes to stress-induced CXCL8 expression by stabilizing CXCL8 mRNA [87]. Numerous *in vitro* studies have demonstrated MAPK pathway inhibitors attenuate stress-induced CXCL8 expression in many cell types. Inhibition of p38 MAPK by the pyridylimidazole analogue, SB203580, has been shown to diminish stress-induced expression of CXCL8 in human cell lines [88]. Another inhibitor of p38 MAPK activity, RWJ-67657, has also been shown to suppress stress-induced expression of CXCL8 in HUVEC cells [89]. The ERK pathway inhibitors PD9805 and U0126 have also been shown to attenuate CXCL8 expression [36]. Recently, Bonavia et al. reported that the pharmacological targeting of the JNK pathway with SP600125 also blocks CXCL8 expression in glioma cells [90]. Thus, this evidence indicates that strategies to target MAPK signaling pathways may effectively attenuate CXCL8 expression in malignant cells. Several MAPK inhibitors, in particular p38 MAPK inhibitors, have entered clinical trials for pulmonary disease as well as for cancer therapeutics. Although the majority of these trials have not sought to assess CXCL8 expression levels in related pharmacodynamic biomarker studies, the results from one recent phase II clinical trial assessing the p38 MAPK inhibitor losmapimod (GW856553X) in COPD, reported no significant effects on serum CXCL8 levels [91].

#### 3.1.2. Phosphatidylinositol-3-Kinase (PI3K)/Akt Inhibitors

The PI3K/Akt signaling pathway is another major intracellular signal transduction pathway, activated by a large number of receptor tyrosine kinases [92] and G protein-coupled receptors. Inappropriate activation of the PI3K pathway (through mutation, loss of negative regulators, or increased cognition of growth factor stimuli), has been reported in a wide range of cancers, making targeting this pathway of considerable interest [93]. Several laboratories have demonstrated a role for this pathway in the regulation of CXCL8 expression [40,94], while we have also shown that loss of the native phosphatidyl-inositol-3-kinase/Akt signaling inhibitor, the tumor suppressor gene PTEN, results in a selective increase in CXCL8 signaling *in vitro* and *in vivo* [14]. This increase in CXCL8 signaling sustains the growth and survival of the PTEN-depleted prostate epithelium. Although the precise mechanisms by which PI3K signaling can regulate CXCL8 remain unclear, Fernandez *et al*. suggest that this may be downstream of p38 MAPK and EGFR activation [95]. Several studies have demonstrated the inhibition of CXCL8 expression by PI3K inhibitors *in vitro*; LY294002 [94,96] and wortmanin [94] can effectively down-regulate TNFα-induced CXCL8 expression in liver cells, while other agents including GDC-0941, BEZ-235 and SHBM1009 down-regulate EGF-stimulated CXCL8 expression in NSCLC cells [97]. Although numerous PI3K inhibitors have also entered clinical trials, none of these yet propose to measure CXCL8 levels as potential pharmacodynamic endpoints or predictive marker of therapeutic response.

The central importance of signal transduction pathways to the development and survival of tumors has made them an attractive target for cancer drug discovery. However, despite the large numbers of MAPK and PI3K pathway inhibitors that have entered clinical trials, these have demonstrated relatively poor success as single agents. Adaptive drug resistance, whereby tumor cells develop mechanisms to resist the anti-tumor effects of these pathway inhibitors, has emerged as a major reason for the failure of such approaches [98]; in a recent study Britschgi *et al*. (2012) proposed that the up-regulation of CXCL8 signaling, in a JAK-dependent manner, may be one such mechanism by which some cancer cells develop resistance to the PI3K inhibitor, BEZ235 [99]. Thus a combined approach, whereby both MAPK and PI3K pathways are concurrently targeted may be the most successful approach to down-regulate signaling-induced CXCL8 gene expression in malignant cells.

### 3.2. NFκB Inhibitors

NFκB is a pro-inflammatory transcription factor, responsible for the regulation of more than 500 target genes [100]. The reciprocal activation between NFκB and inflammatory cytokines makes NFκB an important factor not only for inflammation, but also for cancer development and progression [101]. In particular, NFκB contributes to the initiation and progression of a wide variety of human cancers, through the regulation of genes that are involved in angiogenesis (e.g., CXCL8, VEGF), proliferation (e.g., cyclin D1), evasion of apoptosis (e.g., Bcl-2) and metastasis (e.g., MMP9) [101]. Several studies have demonstrated constitutive NFκB activity in human prostate cancer cell lines and xenografts [102,103,104]. Constitutive NFκB activation, along with the associated increased expression of anti-apoptotic genes, is implicated in the resistance of tumor cells to chemotherapy and radiotherapy while inhibition of NFκB activity sensitizes prostate and colorectal cancer cells to chemotherapy-induced apoptosis [79]. Moreover, activation of NFκB has been shown to be an important promoter of treatment-induced and environmental stress-induced CXCL8 and CXCL8 receptor expression and to contribute to CXCL8-promoted survival and chemoresistance [78]. Therefore, given the central importance of NFκB in the regulation of CXCL8, NFκB-targeted therapeutics may have the desired effect of indirectly targeting CXCL8 signaling. Indeed, our previous *in vitro* studies have demonstrated that CXCL8 expression and signaling may indeed be attenuated by strategies designed to target NFκB [78].

The central role of NFκB in tumor development and progression makes it an attractive therapeutic option. However, poor understanding of the roles of specific NFκB subunits in different stages of cancer progression, along with the current lack of compounds capable of specifically targeting distinct NFκB subunits have hampered advances in this area. Investigations have therefore focused on developing agents that indirectly regulate NFκB, by targeting its post-translational modification including methylation, acetylation, phosphorylation and ubiquitination. Activated NFκB translocates to the nucleus to activate target gene expression [100], and as such the Iκkinase (IκK) has been a major target to prevent its activation [105]. However, although a considerable number of IκK inhibitors have been investigated in pre-clinical models [106], to date none of these have entered clinical trials. Alternatively, proteasome inhibitors regulate the degradation of IκB and hence inhibit NFκB activity. In particular, bortezomib has been shown to reduce tumor growth in xenograft models [107,108] and has been successfully used in first-line treatment in multiple myeloma and mantle cell lymphoma. Bortezomib has also been shown to inhibit proliferation and CXCL8 secretion *in vitro* in bladder [109] and prostate cancer cell lines [110]. A new-generation of proteasome inhibitors including carfilzomib have recently been approved for clinical use but their effects on CXCL8 expression and signaling remain to be determined.

The polyphenol curcumin (diferuloylmethane) is a principal component of turmeric, and demonstrates potent anti-inflammatory and anti-oxidant properties. The effects of curcumin are mediated through the regulation of various transcription factors, in particular NFκB. Curcumin inhibits NFκB-mediated transcription through the regulation of IκK activity [111] and thus the inhibition of IκK phosphorylation. Since NFκB plays a role in the inhibition of apoptosis, it is unsurprising that, in cancer cells, curcumin inhibits proliferation by causing cell cycles arrest and induces apoptosis. Curcumin has also been shown to inhibit the activation of AP-1, through both its ability to suppress JNK activation [112] and through direct interaction with the AP-1-DNA binding site [113], as well as the activation of various STATs [114]. Downstream of these transcription factors, the effect of curcumin is to repress the expression of pro-inflammatory cytokines (e.g., TNF-α), interleukins (e.g., IL-6) and chemokines (e.g., CXCL8) [115,116]. The ability of curcumin to inhibit the synthesis and signaling of CXCL8 was first demonstrated by Hidaka *et al*. in a study conducted in pancreatic cancer cell lines. This was shown to be dependent on the inhibition of NFκB activity [117]. Given the central role of CXCL8 in many of the hallmarks of cancer, it is likely that the inhibition of CXCL8 signaling is a contributing factor to the effects of curcumin on cancer cell survival, angiogenesis and metastasis, as well as the reported ability of curcumin to sensitize tumor cells to the effects of chemotherapy and radiotherapy [114].

The glucocorticosteroid dexamethasone is an anti-inflammatory agent used in the treatment of respiratory tract conditions such as asthma and cystic fibrosis. In this context, it has been known for many years that dexamethasone can decrease CXCL8 expression by airway epithelial cells [118,119]. Although dexamethasone can regulate NFκB activity [120,121], its effects on CXCL8 expression by airway epithelial cells may be independent of NFκB. Indeed, destabilization of the CXCL8 mRNA transcript [119] and signaling through the P38 MAPK pathway have also been reported to play an important role in the dexamethasone-regulated expression of CXCL8 in airway epithelial cells [122]. Dexamethasone has also been shown to suppress CXCL8 expression by lung cancer cells [123]; in a recent study Chen *et al*. demonstrated that the up-regulation of CXCL8 expression by lung cancer cells following exposure to infiltrating macrophages could be attenuated by administration of dexamethasone. Glucocorticoids such as dexamethasone have also been shown to be effective in the treatment of castrate resistant prostate cancer [124], with a biochemical PSA response of up to 49% reported. We have shown that dexamethasone reduces the synthesis and secretion of CXCL8 in prostate cancer cell line models through the capacity to decrease NFκB activity in aggressive prostate cancer cells [125]. Moreover, we have also demonstrated that dexamethasone decreases docetaxel-induced CXCL8 secretion from prostate cancer cells and that *in vivo*, the addition of dexamethasone potentiates the anti-angiogenic activity of docetaxel [126], consistent with the hypothesis that the anti-tumor effects of dexamethasone may be mediated, in part, by the inhibition of stress-induced CXCL8 signaling.

### 3.3. NSAIDS

Non-steroidal anti-inflammatory drugs (NSAIDs) such as aspirin, ibuprofen and sulindac are primarily used for the treatment of acute or chronic inflammatory conditions, primarily due to their ability to inhibit cyclooxygenase (COX) activity. The use of NSAIDs in these conditions may also reduce CXCL8 expression; in *in vivo* studies of arthritis, López-Armada *et al*. demonstrated a decrease in local expression of the pro-inflammatory chemokines CXCL8 and CCL2 following treatment with NSAIDs [127]. Evidence suggests that chronic administration of NSAIDs may also play a role in the prevention of cancer development and/or recurrence. A landmark study in 1997 demonstrated the ability of low-dose aspirin to drastically reduce the risk of developing colon cancer [128]. Subsequent randomized trials have also shown that use of NSAIDs is associated with reduced risk of developing colon, breast, prostate and lung cancers [129]. The chemopreventive properties of NSAIDs have been attributed to their ability to induce apoptosis. Although the precise mechanisms by which NSAIDs prevent cell death remain unclear, this is likely to be through COX-dependent and COX-independent mechanisms.

Recently, pre-clinical studies have demonstrated that phospho-modified ibuprofen (p-ibuprofen) plays a preventative role in the development of colorectal cancer, through the inhibition of NFκB activity [130]. Since NFκB plays a major role in the regulation of CXCL8 and CXCL8 receptor expression [78], p-ibuprofen may also indirectly target CXCL8 signaling, through the regulation of CXCL8, CXCR1 and/or CXCR2 expression. Indeed, CXCL8 has been shown to be down-regulated in response to sulindac and has been identified as a mediator of sulindac-induced apoptosis in colorectal cancer cell line models [131], while salicylate-treatment results in the down-regulation of CXCL8 expression in the macrophage-like cell line THP-1 [132]. Emerging evidence highlights the importance of the associated microenvironment in the growth and survival of tumor cells; therefore strategies to target the inflammatory signaling associated with not only tumor cells but also tumor-associated stromal cells will play an important role in developing therapeutic strategies.

## 4. Direct Targeting

There is an abundance of evidence supporting the validity of targeting CXCL8-CXCR1/2 signaling in cancer. In general, there are multiple means of trying to affect this. For example, CXCL8 itself may be targeted with the use of a neutralizing antibody or alternatively, in an attempt to eliminate the redundancy of chemokine signaling, targeting of the CXCR1 and/or CXCR2 receptors may be attempted using neutralizing antibodies, small molecule antagonists, or peptide-derived inhibitors. Less conventional methods of receptor targeting, such as targeted siRNA nanoparticles will also briefly be discussed. Each of these strategies will be addressed individually, using examples of pre-clinical cancer studies and a number of clinical trials that have been performed using such agents in the treatment of inflammatory diseases.

### 4.1. CXCL8 Neutralizing Antibodies

Multiple studies have used CXCL8 neutralizing antibodies to reduce tumor burden in mouse models, the results of which have been mainly attributed to the inhibition of CXCL8’s angiogenic properties. For example, anti-CXCL8 antibodies have been used to impede the growth of PC3 tumors in SCID mice by reducing angiogenic signaling [59] and have been shown to diminish angiogenesis in orthotopic prostate carcinomas in NOD-SCID mice, as a result of impaired neutrophil infiltration [133].

Multiple anti-CXCL8 antibodies have been used in clinical trials for the treatment of inflammatory diseases. ABX-IL8, a fully humanized monoclonal anti-CXCL8 antibody produced by Abgenix using XenoMouse technology has been assessed in clinical trials for rheumatoid arthritis, psoriasis and chronic obstructive pulmonary disease (COPD). Despite being found to be safe and well tolerated, ABX-IL8 failed to significantly reduce patient symptoms in phase II trials for rheumatoid arthritis and psoriasis [134]. Nevertheless, ABX-IL8 continued to be pursued for other indications, including COPD and malignant melanoma. In 2004, ABX-IL8 was assessed in phase II clinical trials of COPD where it was found to reduce the severity of dyspnea relative to placebo [135]. The antibody infusions were well tolerated, with no significant differences in health status or adverse events between treatment and placebo groups. Although, to our knowledge, there are no clinical trial results for this antibody in metastatic melanoma, ABX-IL8 did show promising results in pre-clinical melanoma studies, reducing tumor growth and angiogenesis in A375SM and TXM-13 xenografts in nude mice [136].

Another anti-CXCL8 antibody, HuMab 10F8, has been shown to reduce disease activity of palmoplantar pustulosis, a chronic inflammatory skin disease [137]. Again, the anti-CXCL8 antibody was well tolerated, with no serious adverse events attributed to treatment, with the most frequently reported mild/moderate adverse events including nausea, nasopharyngitis and headache. Notably, HuMab 10F8 was found to cause no immunogenicity or evidence of cytokine release syndrome.

These studies highlight the potential for the use of a CXCL8 neutralizing antibody in human cancers, given that they were found to be well tolerated and capable of reducing disease activity in certain inflammatory diseases. However, the use of anti-CXCL8 antibodies does not account for the redundant nature of CXC-chemokine signaling, wherein targeting the CXCL8 ligand alone would fail to inhibit the activity of the other CXC-chemokines such as CXCL1, CXCL5 or CXCL6 which are capable of activating the CXCR2 receptor, and which there is evidence may also be up-regulated within the tumor microenvironment [138,139,140]. Anti-CXCL8 antibodies would also fail to inhibit any tumor-promoting MIF signaling, which has previously been demonstrated to potentiate growth of PC3 tumors *in vivo* [141]. Therefore, targeting the CXCL8 receptors is likely to be more efficacious than neutralizing CXCL8 alone.

### 4.2. CXCR1/2 Neutralizing Antibodies

Neutralizing antibodies may also be used to target CXCR1 and CXCR2, preventing ligand binding at the extracellular domain. A collection of *in vitro* studies have illustrated the anti-tumor potential of CXCR1/2 neutralizing antibodies. Blockade of CXCR1 via neutralizing antibody has been shown to inhibit CXCL8-induced proliferation of small-cell lung cancer (SCLC) cell lines [142]. However, neutralization of CXCR2 did not significantly reduce SCLC proliferation, highlighting the independent roles of signaling induced downstream of the two receptors. CXCR1 and CXCR2 neutralizing antibodies have also been used to inhibit cytoskeletal reorganization of endothelial cells, with the early response inhibited by CXCR1 blockade, and cell retraction during the later phase inhibited by CXCR2 blockade [33].

*In vivo*, CXCR2 neutralizing antibodies have been shown to inhibit the progression of premalignant alveolar lesions, where they were found to induce apoptosis in the endothelial cells located within these lesions [12]. CXCR2 neutralizing antibodies have also been shown to attenuate lung neovascularization following left pulmonary artery ligation in mouse models, demonstrating the ability of CXCR2 neutralizing antibodies to impede angiogenesis [143]. Farooq *et al*. used anti-CXCR2 antiserum to reduce the incidence of dextran sodium sulfate (DSS)-induced colitis in mice, attributed to a reduction in polymorphonuclear neutrophil (PMN) infiltration [76]. Moreover, in an orthotopic model of pancreatic cancer, Matsuo *et al*. showed that polyclonal anti-mouse CXCR2 neutralizing antibodies were able to significantly reduce tumor volume and microvessel density [61]. Despite promising *in vivo* results demonstrating the ability of CXCR2 neutralization to induce apoptosis, impede angiogenesis and reduce inflammation-associated PMN infiltration, CXCR2 neutralizing antibodies have not yet been taken forward to clinical trials.

### 4.3. Small Molecule CXCR1/2 Antagonists

Small molecule antagonists against CXCR1 and/or CXCR2 have been designed by a number of pharmaceutical companies to non-competitively inhibit receptor activation. Characteristics of the CXCR1/2 antagonists developed by each of these companies will be reviewed individually, giving a few examples from each.

#### 4.3.1. Dompé

Reparixin ((2R)-2-[4-(2-methylpropyl)phenyl]-*N*-(methylsulfonyl)propanamide), formerly known as repertaxin, is a small molecule allosteric antagonist developed by the Italian pharmaceutical company Dompé. It is an acylmethanesulfonamide derivative, originally designed to attenuate CXCL8-induced chemotaxis of neutrophils to sites of inflammation, for which it has an IC_50_ of 1 nM [144]. Reparixin is approximately 400-fold more selective for CXCR1 than CXCR2, with its mechanism of action involving binding at the transmembrane region of CXCR1, where it allosterically inhibits agonist-induced receptor activation and induction of downstream signaling, without directly affecting CXCL8 binding affinity or the level of receptor cell surface expression [145]. A pre-clinical study performed by Ginestier *et al*. showed that reparixin was capable of targeting breast cancer stem cells in xenograft models, resulting in a reduction of tumor growth and metastasis, both when administered as a monotherapy, or when given in combination with docetaxel [146].

Currently, patients are being recruited for a phase 1b study where reparixin will be administered in combination with paclitaxel in HER-2 negative breast cancer. Patients in this study receive 3 days of oral reparixin tablets, 3 times a day, followed by a cycle of combined paclitaxel (80 mg/m^2^/week) and reparixin 3 times a day for 21 days, in three different dosages (400 mg, 800 mg and 1,200 mg). The second cohort of the study has now been completed, with no reparixin-related toxicity observed [147]. Recruitment is also underway for a phase III trial evaluating reparixin treatment in patients with type I diabetes who have undergone transplantation of insulin-producing islets [148]. The results of the phase II study showed that reparixin-treated patients experienced improved transplant outcome and decreased insulin requirement, with no reparixin-related adverse events reported [149]. Reparixin is currently being evaluated in clinical trials in a number of other indications, including kidney [150] and lung transplantation [151].

Dompé have also developed a number of related compounds, including DF2162 (4-[(1*R*)-2-amino-1-methyl-2-oxoethyl]phenyl trifluoromethane sulfonate) which is capable of inhibiting both CXCR1 and CXCR2. DF2162 has shown promising results in pre-clinical studies of adjuvant-induced polyarthritis [152] and bleomycin-induced pulmonary inflammation and fibrosis [153], but is yet to be assessed in clinical trials. Again, it is of note that these animal studies would have inhibited KC rather than CXCL8 signaling through CXCR1/2.

#### 4.3.2. Schering-Plough

Schering-Plough have developed a cyclobutenedione compound, SCH527123 [2-hydroxy-*N*,*N*-dimethyl-3-[[2-[[1(*R*)-(5-methyl-2-furanyl)propyl]amino]-3,4-dioxo-1-cyclobuten-1-yl]amino]benzamide], a potent intracellular allosteric CXCR1/2 antagonist [154]. SCH527123 binds with micromolar affinity to CXCR1 and picomolar affinity to CXCR2 and is therefore, in terms of its therapeutic use, CXCR2-selective [155]. SCH527123 has been used in a pre-clinical xenograft model of colorectal cancer where SCH527123 treatment reduced tumor growth and microvessel density [82]. In addition, the administration of SCH527123 in combination with oxaliplatin resulted in greater reductions in cell proliferation, tumor growth and angiogenesis relative to the effects of either agent alone. An additional pre-clinical study showed that SCH527123 and a related compound SCH479833 could inhibit the development of liver metastasis from human colon cancer cells implanted in the spleen of nude mice, again a response attributed to decreased neovascularization and increased tumor cell apoptosis [156].

A clinical study has been undertaken by Holz and colleagues to demonstrate the ability of oral SCH527123 to reduce sputum neutrophil counts following ozone challenge in healthy subjects, relative to prednisolone or placebo [157]. Oral SCH527123 was found to be safe and well tolerated. A similar study in patients with COPD showed that SCH527123 could significantly reduce sputum neutrophils by 47% compared to placebo, with the frequency of adverse events similar between treatment and placebo groups [158]. Furthermore, a clinical trial assessing the safety and efficacy of SCH527123 in the treatment of patients with severe asthma showed a reduction in sputum neutrophils and a modest improvement in asthma control, with no treatment-induced adverse events observed [159].

#### 4.3.3. GlaxoSmith Kline (GSK)

GSK was one of the first pharmaceutical companies to develop selective CXCR2 antagonists. SB225002 (*N*-(2-bromophenyl)-*N*′-(2-hydroxy-4-nitrophenyl)urea) is a phenol-containing diarylurea small molecule antagonist with >150 fold selectivity for CXCR2 over CXCR1 and an IC_50_ of 22 nM [160,161]. SB225002 has been used *in vivo* to reduce acute experimental colitis in BALB/c mice [162], but did not reach clinical trials as development was ceased due to undesirable pharmacokinetic properties [161]. However, further research led to the discovery that the insertion of a sulfonamide group into the phenol ring greatly reduced metabolic clearance [163]. This led to the development of a novel GSK CXCR2 antagonist, SB656933 (*N*-(2-chloro-3-fluorophenyl)-*N*′-[4-chloro-2-hydroxy-3-(piperazin-1-ylsulfonyl) phenyl]-urea), which binds to CXCR2 with an IC_50_ of 22 nM [162]. SB656933 has been used in a clinical study to reduce ozone-induced airway inflammation in humans, and was found to be well tolerated at all doses [164]. SB656933 has also been used in a clinical trial for the treatment of cystic fibrosis [165]. Patients showed trends towards improvement in sputum inflammatory biomarkers. However, there were no changes in lung function or respiratory symptoms. SB656933 was generally well tolerated with headache the most frequently reported adverse event across all groups. There were no cases of neutropenia and no change in bacterial colonization of sputum throughout the duration of the study. SB656933 has also recently been used in a clinical trial for the treatment of ulcerative colitis [166], with results remaining unpublished to date.

#### 4.3.4. AstraZeneca

AstraZeneca are responsible for the development of a number of fused pyrimidine series-based CXCR2-selective antagonists [167]. One such agent from this series, AZD8309, for example, has been used in phase I clinical trials for COPD and phase II for rheumatoid arthritis [168]. AZD8309 was well tolerated in a study investigating the ability of CXCR2 inhibition to reduce LPS-induced neutrophil recruitment in the upper airways of healthy subjects [169]. However, the development of AZD8309 was reportedly ceased by AstraZeneca in 2007, when they published on the development of a series of substituted thiazolo[4,5-*d*]pyrimidine novel CXCR2 antagonists [170].

A novel AstraZeneca CXCR2 antagonist, AZD5069 (N-[2-[[(2,3-difluoropheny)methyl]thio]-6-{[(1R,2S)-2,3-dihydroxy-1-methylpropyl]oxy}-4-pyrimidinyl]-1-azetidinesulfonamide), was shown to be well tolerated throughout a phase I study in healthy subjects [171]. AZD5069 was subsequently entered into a number of phase II studies for indications including COPD, bronchiectasis and asthma. AZD5069 was administered as oral capsules (50 or 80mg) twice daily for the treatment of COPD in a 4-week, phase II study [172]. Primary outcome measures for this study were safety and tolerability, with secondary outcome measures including pharmacokinetics and neutrophil counts. An additional phase II study has assessed the use of AZD5069 for the treatment of bronchiectasis [173]. In this study, patients were administered oral AZD5069 twice daily, for 28 days, with primary outcome measures including neutrophil counts, and secondary outcome measures including signs and symptoms of bronchiectasis, safety and tolerability, inflammatory markers, and pharmacokinetics. However, the results of these phase II studies are yet to be released. Currently, AstraZeneca are recruiting participants for a phase II study for AZD5069 in the treatment of uncontrolled persistent asthma [174].

In summary, a range of CXCR1 and/or CXCR2 small molecule allosteric inhibitors are under development by a number of major pharmaceutical companies (Table 1). However, the majority of these antagonists have only been used in clinical trials for the treatment of inflammatory conditions, and many of them are yet to enter clinical studies for the treatment of human cancer. Results of the clinical study using a reparixin-paclitaxel combination in HER-2 negative breast cancer may highlight the potential for the use of CXCR1/2 small molecule antagonists as a viable strategy for the treatment of cancer, most likely in combination with established chemotherapies.

### 4.4. Pepducin CXCR1/2 Inhibitors

Another approach to CXCR1/2 inhibition involves the use of pepducins as peptide inhibitors. Pepducins are composed of a lipid moiety (e.g., palmitate, myristate or lithocholic acid) which is attached at the N-terminal of a synthetic peptide corresponding to a specific amino acid sequence, typically 10–20 amino acids in length, from one of the intracellular loops (i1, i2 or i3) or the C-terminal tail (i4) of the GPCR of interest [175]. The lipid moiety permits translocation across the plasma membrane and is believed to anchor the pepducin at the intracellular face of the plasma membrane, increasing molarity in the proximity of the target receptor, where the amino acid sequence can inhibit activation of signaling by interrupting the interaction between the intracellular loops of the receptor and its G protein. CXCR1/2 pepducins have been designed to target the first (i1) and third (i3) intracellular loops of CXCR1 and CXCR2, which are identical in sequence permitting dual targeting of signaling from both these receptors.

**Table 1:** Table summarizing the best characterized CXCR1/2 small molecule antagonists available from a range of pharmaceutical companies, and the pre-clinical cancer studies and clinical trials in other inflammatory conditions in which they have be utilized.

Company	Dompé		Schering-Plough		GlaxoSmith Kline		AstraZeneca	
**Antagonist**	CXCR1	CXCR2	CXCR1	CXCR2	CXCR1	CXCR2	CXCR1	CXCR2
	Reparixin			SCH527123		SB225002 SB656933 *		AZD8309 AZD5069 *
	DF2162 *							
**Cancer pre-clinical studies**	Breast cancer xenografts		Colorectal cancer xenografts		Colitis			
**Clinical trials**	Diabetes (islet cell transplantation) * Breast cancer*		Ozone-induced neutrophilia COPD Asthma		Ozone-induced airway inflammation * Cystic fibrosis * COPD * Ulcerative colitis *		COPD Rheumatoid arthritis COPD * Bronchiectasis * Asthma *	

* depicts corresponding antagonist and study; *Italics* - trial currently recruiting/underway.

A pre-clinical study by Kaneider *et al*. showed that pepducins designed against either the i1 or i3 intracellular loops of CXCR1/2 are capable of reversing multiple processes associated with systemic inflammatory response syndrome in septic mice, including a reduction in neutrophil chemotaxis and liver damage, and protection from thrombocytopenia [176]. This study showed that X1/2pal-i3 pepducins could completely inhibit neutrophil migration into the peritoneal cavity with an IC50 value of 0.03mg/kg. Moreover, an ovarian cancer study demonstrated the ability of X1/2pal-i3 to attenuate CXCL1/CXCL8-induced endothelial cell proliferation and tube formation in vitro [177]. Additionally, this study showed attenuation of angiogenesis and ovarian tumor growth in mice treated with X1/2pal-i3. Mice were treated with 5 mg/kg/day in the seven (angiogenesis model) or thirty (xenograft model) days prior to sacrifice. Furthermore, Jamieson et al. showed that CXCR1/2-targeted pepducins could inhibit adenoma formation in APC(Min/+) mice, attributed to a reduction in neutrophil recruitment during tumor-inducing inflammation [77]. In this study, 35 day old APC(Min/+) mice were given daily subcutaneous injections of 2.5 mg/kg X1/2pal-i3 pepducins or control X1/2pal-i3CONT pepducin dissolved in sterile saline until the day of sacrifice. To date, pepducin inhibitors have not yet entered any clinical studies. Therefore, their safety and efficacy in the treatment of human disease is currently unknown.

*4.5. siRNA Strategies*

A recent clinical study by Davis et al. showed for the first time, that siRNA nanoparticles were able to induce RNA interference (RNAi) in human cancer patients, without eliciting an interferon response [178]. They were able to demonstrate successful knock down of mRNA and protein expression of their specific gene of interest, RRM2. The siRNA used in the study, although administered systemically, was delivered specifically to the tumor site via targeted nanoparticles. These siRNA nanoparticles consisted of a linear cyclodextrin-based polymer, a human transferrin protein-targeting ligand (targeting the transferrin receptors of tumor cells), a hydrophilic polymer for stability, and siRNA designed against RRM2. Crucially, they showed that the amount of intracellular nanoparticles correlated with the dose of nanoparticles administered to the patient. A handful of other studies have also shown promising results with the use of targeted siRNA nanoparticles [179,180]. Although CXCL8 or CXCR1/2 siRNAs have not yet been used clinically, a pre-clinical orthotopic ovarian cancer model has illustrated anti-tumor effects upon silencing of CXCL8 gene expression using liposome-encapsulated siRNA [181]. Further research and development of targeted siRNA nanoparticles and their pharmacokinetic profiles may lead to an increase in the use of RNAi-based strategies in a clinical setting, providing another possible method for inhibition of CXCL8-CXCR1/2 signaling in cancer.

## 5. Translational Issues

A significant number of *in vitro* and *in vivo* pre-clinical studies can now be cited to support the importance of CXCL8-CXCR1/2 signaling in promoting tumor progression, via promotion of multiple hallmarks of cancer. Attenuating CXCL8-CXCR1/2 signaling (via neutralizing antibodies, small molecule antagonists and pepducins) has been shown to have major inhibitory effects on tumor growth, angiogenesis and tumor dissemination. Despite this extensive pre-clinical literature, there has been limited exploitation of this knowledge in clinical trials aimed at evaluating anti-CXCL8 or CXCR1/2 inhibitors as anti-cancer therapeutics. The reparixin-paclitaxel combination study in HER-2 negative breast cancer represents the first major clinical study in malignant disease. Clinical trials using CXCL8 or CXCR1/2 inhibitors in treatment of other inflammatory diseases (including COPD, rheumatoid arthritis, asthma, psoriasis, palmoplantar pustulosis), type I diabetes, or to prevent transplant rejection have shown that neutralizing antibodies or small molecule antagonists are inherently safe for use in patients, with no significant adverse events detected relative to placebo-treated groups. However, the remaining caveat to their use in oncology trials is whether such treatments may be safe in cancer patients who may already have a compromised immune system and be suffering from chemotherapy-induced neutropenia.

With our increasing understanding of the extensive molecular heterogeneity of cancers, and the current trajectory of the field towards the prosecution of precision medicine, one of the important considerations regarding the exploitation of anti-CXCL8-CXCR1/2 therapeutics will be establishing which tumors are susceptible to such inhibitors. Work from our laboratory has determined that the induction of CXCL8 signaling plays an important role in sustaining the viability of PTEN-deficient prostate carcinomas [14]. PTEN haplo-insufficiency occurs in approximately 70–80% of primary tumors and is associated with the early progression of the disease, while homozygous deletion of PTEN is associated with advanced stage tumors and the development of metastatic disease [182]. Therefore, pre-clinical evidence suggests that evaluating anti-CXCL8 therapeutics would be more clinically relevant in patients with confirmed PTEN^+/−^ or PTEN^−/−^ prostate tumors. CXCL8 signaling is also known to be promoted downstream of KRas mutations [11,12], suggesting that enrichment of lung, pancreatic or colorectal tumors harboring KRas mutations may be more responsive to anti-CXCL8 therapeutics. Recent studies have also indicated a role of CXCR2 signaling in promoting the early tumor development of APC-deficient colorectal cancer, wherein polyp formation was significantly depleted by the inhibition of this signaling [77]. Accordingly, the use of anti-CXCL8 signaling inhibitors could be used as a chemo-preventative strategy in high-risk patient groups. More extensive pre-clinical research will be required to map all of the appropriate genetic backgrounds in which these anti-CXCL8 therapeutics can be exploited across all of the major solid tumor types.

One further consideration in exploiting anti-CXCR1/2-targeted therapies in oncology is whether the therapy is best used as a chemo-modulator or sensitizing agent to radiotherapy. Pre-clinical studies from numerous groups have demonstrated the ability of CXCR1/2 inhibitors to sensitize multiple tumor models to a plethora of clinically-relevant chemotherapies. The inhibition of autocrine CXCL8 signaling has been shown to sensitize PTEN-deficient or p53-mutant cancer cells to DNA-damaging agents, anti-metabolites or androgen receptor-targeting strategies [50,78,79,81], while the anti-angiogenic activity of the CXCL8-targeting strategies have been shown to augment anti-angiogenic responses elicited by docetaxel in ovarian and prostate cancer models [126,181]. Accordingly, the optimal use of anti-CXCR1/2 therapy may need to consider a number of distinct parameters that include the genetic background of the tumor, the preferred type of response that is required in the context of current tumor stage and finally, what is the preferred clinical agent with which it can be combined in order to induce the maximal response. Much research remains to be conducted, including performing retrospective analysis of tumor material to demonstrate the clinical relevance of this pathway to disease progression of genetically-discrete populations of solid tumors. When matched with the demonstration of the effects in appropriate models of the living environment of tumors such as patient-derived xenografts, the potential of using anti-CXCL8-CXCR1/2-targeting therapeutics in the clinical management of cancer may become a true and widely exploited reality.

## 6. Conclusions

There is an extensive body of evidence to support the use of a CXCR1/2-targeted therapy in the treatment of human cancers. Clinical studies using such interventions for the treatment of inflammatory diseases have shown that the armory of CXCR1/2 antagonists and neutralizing antibodies that have been developed are safe for use in human patients. As pre-clinical research continues to uncover the relevance of this pathway to the progression of multiple tumor types, and to increasingly demonstrate its importance to aggressive tumor hallmarks in specific genetic backgrounds, there is compelling evidence to begin to evaluate anti-CXCL8 signaling inhibitors in human cancer. Potent effects of these therapeutics as anti-angiogenics and chemo-modulators are expected on the basis of multiple tumor models. More specifically, tumors harboring specific genetic aberrations such as PTEN loss, or KRas activation, would be expected to show exquisite sensitivity to anti-CXCL8 targeting therapeutics. Careful consideration of the capacity of these therapeutics to enhance several treatment modalities such as DNA-damage therapy may also assist in accelerating their exploitation in “tailored” or “personalized” cancer therapy.
